# Adaptive enrichment trials: What are the benefits?

**DOI:** 10.1002/sim.8797

**Published:** 2020-11-26

**Authors:** Thomas Burnett, Christopher Jennison

**Affiliations:** ^1^ Department of Mathematics and Statistics Lancaster University Lancaster UK; ^2^ Department of Mathematical Sciences University of Bath Bath UK

**Keywords:** adaptive designs, adaptive enrichment, Bayesian optimization, phase III clinical trial, population enrichment

## Abstract

When planning a Phase III clinical trial, suppose a certain subset of patients is expected to respond particularly well to the new treatment. Adaptive enrichment designs make use of interim data in selecting the target population for the remainder of the trial, either continuing with the full population or restricting recruitment to the subset of patients. We define a multiple testing procedure that maintains strong control of the familywise error rate, while allowing for the adaptive sampling procedure. We derive the Bayes optimal rule for deciding whether or not to restrict recruitment to the subset after the interim analysis and present an efficient algorithm to facilitate simulation‐based optimisation, enabling the construction of Bayes optimal rules in a wide variety of problem formulations. We compare adaptive enrichment designs with traditional nonadaptive designs in a broad range of examples and draw clear conclusions about the potential benefits of adaptive enrichment.

## 1 INTRODUCTION

Consider a Phase III trial in which it is believed a certain subset of patients will respond particularly well to the new treatment. We wish to test for a treatment effect in both the pre‐identified subpopulation and the full population. Such multiple testing can be conducted using a closed testing procedure to control the familywise error rate (FWER).^1^ In an adaptive enrichment design, if interim data suggest it is only the subpopulation that benefits from the new treatment, recruitment in the second half of the trial is restricted to the subpopulation. This increase in recruitment from the subpopulation is referred to as “enrichment” of the sampling rule.

We develop and assess designs which use a closed testing procedure with Simes' method^2^ to test the intersection hypothesis and a weighted inverse normal combination test^3^, ^4^, ^5^ to combine data from the two stages of the trial. We show that the resulting testing procedure controls the FWER, whatever rule is used to decide when enrichment should occur. This allows us to seek the enrichment rule which is optimal for a specified criterion. We shall follow the approach presented by Burnett,^6^ defining a gain function that reflects the value of the outcome of the trial and a prior distribution for the treatment effects in the subpopulation and full population. The optimal decision at the interim analysis is that which maximises the expected gain with respect to the posterior distribution of the treatment effects, given current data. Since we use simulation in constructing the Bayes optimal decision rule for an adaptive design, our approach has the potential to be computationally expensive. We present an efficient algorithm for deriving this decision rule that significantly reduces the calculation required: using our methods, designs can be derived and tested in a matter of minutes on a laptop or PC.

In previous work on adaptive enrichment designs, Brannath et al^7^ followed a Bayesian approach, assuming an uninformative prior for treatment effects. They determined the enrichment decision by comparing the posterior predictive probabilities of rejecting each hypothesis at the end of the trial with certain user‐defined thresholds. Götte et al^8^ considered families of enrichment rules defined in terms of linear combinations of the two treatment effect estimates or the conditional power to reject each hypothesis. They defined the “correct decision” at the interim analysis for given true values of the treatment effects and searched within their families of enrichment rules to maximise a weighted combination of the probabilities of a correct decision. Uozomi and Hamada^9^ defined enrichment rules in terms of thresholds for the treatment effect estimates or predictive power for the two hypothesis tests and set these thresholds to optimize a utility function under specific values for the true treatment effects. Our methods are set in a more complete Bayesian decision theoretic framework. The gain function is chosen to summarize the benefits of the final decisions, reflecting the size of population in which the new treatment is proven to be effective and the magnitude of the treatment effect in this population. The decision whether or not to enrich at the interim analysis is informed by both the posterior distribution of treatment effects and the interim estimates or p‐values that will form part of the final hypothesis tests.

Ondra et al^10^ developed Bayes optimal methods in a class of adaptive enrichment designs where FWER is controlled by a Bonferroni adjustment, assuming a 4‐point discrete prior distribution for the two treatment effects. These simplifications allow the optimal enrichment decision rule to be found by maximising an integral, which is computed numerically. The application of Simes tests in our methods reduces conservatism in the testing procedure and the continuous prior distributions are better able to capture investigators' prior beliefs. Although our form of problem requires the use of simulation to find an optimal design, this approach has the advantage of extending very easily to other forms of gain function and multiple testing methods.

Through studying optimal designs, we are able to assess the potential benefits of adaptive enrichment. We have studied a variety of scenarios, drawing comparisons in each case with two nonadaptive designs: sampling the full population throughout the whole study or focusing on the subpopulation at the outset and only recruiting subpopulation patients. We see there are plausible prior distributions for which the adaptive enrichment design is superior to both forms of nonadaptive design. Furthermore, we recognize that investigators may be reluctant to restrict recruitment to the subpopulation from the outset and observe that in situations where this would have been the optimal policy, adaptive enrichment can give substantially higher expected gain than the nonadaptive, full population design.

Our studies also shed light on the underlying reasons for the effectiveness of adaptive designs. The good performance of adaptive designs in the special case of one‐point prior distributions shows efficiency gains can follow from adapting to interim data and the likelihood of eventual rejection of each null hypothesis. With proper prior distributions, one might expect increased knowledge about the true treatment effects at the interim analysis to give adaptive designs a further advantage. However, we find such benefits to be modest: when the prior variance is high, considerable uncertainty about the true treatment effects remains; when the prior variance is low, information about the treatment effects at the interim analysis comes primarily from the prior, not the interim data.

The paper is structured as follows. We formulate the problem in Section 2 and we present methods for controlling FWER and combining data across stages in Section 3. We describe methods for optimising an adaptive design in Section 4, describe two forms of nonadaptive design in Section 5 and present examples in Section 6. We conclude with discussion of the results obtained in our examples.

## 2 PROBLEM FORMULATION

### 2.1 Patient responses

Consider a Phase III trial comparing a new therapy, Treatment A, with a control, Treatment B. Suppose a biomarker‐defined subpopulation is identified before the trial commences and it is thought that biomarker positive patients will respond particularly well to the new treatment. We call the subpopulation of biomarker positive patients ${𝒮}_{1}$ and the complement of this ${𝒮}_{2}$.

We suppose responses are normally distributed with a common variance $\sigma^{2}$ but note that, by large sample theory, distributions of treatment estimates will have the same form for a wide variety of response types. Let $\mu_{A1}$ and $\mu_{B1}$ be the expected responses for patients in ${𝒮}_{1}$ on Treatments A and B, respectively. Similarly, let $\mu_{A2}$ and $\mu_{B2}$ be the expected responses on Treatments A and B for patients in ${𝒮}_{2}$. Letting *X*_*ij*_ denote the response of the *i*th patient in subpopulation ${𝒮}_{j}$ on Treatment A and *Y*_*ij*_ the response of the *i*th patient in ${𝒮}_{j}$ on Treatment B, we have

$$X_{ij}∼N(\mu_{Aj},\sigma^{2}),\;i=1,2,\ldots ,\;j=1,2,$$

and

$$Y_{ij}∼N(\mu_{Bj},\sigma^{2}),\;i=1,2,\ldots ,\;j=1,2.$$

The treatment effects in subpopulations ${𝒮}_{1}$ and ${𝒮}_{2}$ are $\theta_{1}=\mu_{A1}-\mu_{B1}$ and $\theta_{2}=\mu_{A2}-\mu_{B2}$, respectively.

Suppose ${𝒮}_{1}$ represents a fraction λ of the full population. Then, the overall treatment effect in the full population is $\theta_{3}=\lambda \theta_{1}+(1-\lambda )\theta_{2}$. We shall write $\theta =(\theta_{1},\theta_{2})$, noting that θ determines the value of $\theta_{3}$. We assume the investigators are interested in testing *H*_01_: $\theta_{1}\leq 0$ vs $\theta_{1}>0$ and *H*_03_: $\theta_{3}\leq 0$ vs $\theta_{3}>0$. The hypothesis *H*_02_: $\theta_{2}\leq 0$, is not to be tested (although one might require some evidence of a positive treatment effect in *S*_2_ to support approval of the new treatment for the full population when *H*_03_ is rejected). However, the approach we describe can also be applied when enrichment in either *S*_1_ or *S*_2_ is possible, or when there are more than two subpopulations; the key requirement is that the subpopulations and enrichment options are predefined.

### 2.2 Adaptive enrichment trial designs

If the new therapy is beneficial to all patients, we would hope to reject the null hypothesis *H*_03_ and establish that there is an effect in the full patient population. However, if the benefit is restricted to patients in ${𝒮}_{1}$, it would be advantageous to focus on this subpopulation and increase the probability of rejecting *H*_01_. Adaptive enrichment designs aim to balance these two objectives by using interim data to decide whether or not to restrict enrolment in the remainder of the study to ${𝒮}_{1}$ and test only *H*_01_.

We consider trial designs with a single interim analysis that takes place after a fraction τ of the planned sample size has been recruited and responses from these patients have been observed. Initially, patients are recruited from the full population. If, at the interim analysis, results on the new therapy are promising in both ${𝒮}_{1}$ and ${𝒮}_{2}$, recruitment continues across the full population. If, however, the new therapy only appears to benefit patients in ${𝒮}_{1}$, the remainder of the sample size is devoted to ${𝒮}_{1}$. Our objective is to optimize the rule for choosing between these two options in an adaptive enrichment design.

Let *n* be the total number of patients to be recruited. Assuming recruitment from ${𝒮}_{1}$ and ${𝒮}_{2}$ is in proportion to the size of these subpopulations, sample sizes at the interim analysis are λτn in ${𝒮}_{1}$ and (1−λ)τn in ${𝒮}_{2}$. When recruitment continues from the full population, an additional λ(1−τ)n patients are sampled from ${𝒮}_{1}$ and (1−λ)(1−τ)n from ${𝒮}_{2}$. If “enrichment” occurs and only patients from ${𝒮}_{1}$ are recruited after the interim analysis, there will be a further (1−τ)n patients from ${𝒮}_{1}$. We assume that, within each stage of the trial, patients in each subpopulation are randomized equally between Treatments A and B.

In describing the distributions of parameter estimates, it is helpful to define

(1) $$\tilde{ℐ}=\frac{n}{4\sigma^{2}}.$$

Note that a fixed sample size trial with *n* patients divided equally between Treatments A and B would produce an estimate ${\hat{\theta }}_{3}$ with $Var\;({\hat{\theta }}_{3})=4\sigma^{2}/n$, so $\tilde{ℐ}={\left\{Var\;({\hat{\theta }}_{3})\right\}}^{-1}$ represents the Fisher information for $\theta_{3}$ in this case.

Let $m_{11}=\lambda \tau n/2$ and $m_{21}=(1-\lambda )\tau n/2$. Then, in the form of adaptive enrichment design we have described, the first stage yields treatment effect estimates

$$\begin{array}{ll} {\hat{\theta }}_{1}^{\left(1\right)} & ={\hat{\mu }}_{A1}^{\left(1\right)}-{\hat{\mu }}_{B1}^{\left(1\right)}=\frac{1}{m_{11}}\overset{m_{11}}{\underset{i=1}{\sum }}X_{i1}-\frac{1}{m_{11}}\overset{m_{11}}{\underset{i=1}{\sum }}Y_{i1}∼N(\theta_{1},{\left\{\lambda \tau \tilde{ℐ}\right\}}^{-1}),\\ {\hat{\theta }}_{2}^{\left(1\right)} & ={\hat{\mu }}_{A2}^{\left(1\right)}-{\hat{\mu }}_{B2}^{\left(1\right)}=\frac{1}{m_{21}}\overset{m_{21}}{\underset{i=1}{\sum }}X_{i2}-\frac{1}{m_{21}}\overset{m_{21}}{\underset{i=1}{\sum }}Y_{i2}∼N(\theta_{2},{\left\{(1-\lambda )\tau \tilde{ℐ}\right\}}^{-1}) \end{array}$$

and

$${\hat{\theta }}_{3}^{\left(1\right)}=\lambda {\hat{\theta }}_{1}^{\left(1\right)}+(1-\lambda ){\hat{\theta }}_{2}^{\left(1\right)}∼N(\theta_{3},{\left\{\tau \tilde{ℐ}\right\}}^{-1}).$$

The joint distribution of $\left({\hat{\theta }}_{1}^{\left(1\right)},{\hat{\theta }}_{3}^{\left(1\right)}\right)$ is bivariate normal with correlation $\sqrt{\lambda }$.

Suppose that after the initial analysis the trial continues in the full population. Then, setting $m_{12}=\lambda (1-\tau )n/2$ and $m_{22}=(1-\lambda )(1-\tau )n/2$, the second stage data alone yield treatment effect estimates

$$\begin{array}{ll} {\hat{\theta }}_{1}^{\left(2\right)} & ={\hat{\mu }}_{A1}^{\left(2\right)}-{\hat{\mu }}_{B1}^{\left(2\right)}=\frac{1}{m_{12}}\overset{m_{11}+m_{12}}{\underset{i=m_{11}+1}{\sum }}X_{i1}-\frac{1}{m_{12}}\overset{m_{11}+m_{12}}{\underset{i=m_{11}+1}{\sum }}Y_{i1}∼N(\theta_{1},{\left\{\lambda (1-\tau )\tilde{ℐ}\right\}}^{-1}),\\ {\hat{\theta }}_{2}^{\left(2\right)} & ={\hat{\mu }}_{A2}^{\left(2\right)}-{\hat{\mu }}_{B2}^{\left(2\right)}=\frac{1}{m_{22}}\overset{m_{21}+m_{22}}{\underset{i=m_{21}+1}{\sum }}X_{i2}-\frac{1}{m_{22}}\overset{m_{21}+m_{22}}{\underset{i=m_{21}+1}{\sum }}Y_{i2}∼N(\theta_{2},{\left\{(1-\lambda )(1-\tau )\tilde{ℐ}\right\}}^{-1}), \end{array}$$

and

$${\hat{\theta }}_{3}^{\left(2\right)}=\lambda {\hat{\theta }}_{1}^{\left(2\right)}+(1-\lambda ){\hat{\theta }}_{2}^{\left(2\right)}∼N(\theta_{3},{\left\{(1-\tau )\tilde{ℐ}\right\}}^{-1}).$$

Again, the pair of estimates $\left({\hat{\theta }}_{1}^{\left(2\right)},{\hat{\theta }}_{3}^{\left(2\right)}\right)$ is bivariate normal with correlation $\sqrt{\lambda }$.

Alternatively, suppose the trial is enriched and only subpopulations ${𝒮}_{1}$ is sampled in the second stage. Then, setting ${\tilde{m}}_{12}=(1-\tau )n/2$, the new data yield the estimate

$${\hat{\theta }}_{1}^{\left(2\right)}=\frac{1}{{\tilde{m}}_{12}}\overset{m_{11}+{\tilde{m}}_{12}}{\underset{i=m_{11}+1}{\sum }}X_{i1}-\frac{1}{{\tilde{m}}_{12}}\overset{m_{11}+{\tilde{m}}_{12}}{\underset{i=m_{11}+1}{\sum }}Y_{i1}∼N(\theta_{1},{\left\{(1-\tau )\tilde{ℐ}\right\}}^{-1})),$$

and no estimate of $\theta_{3}$ is available.

## 3 ACHIEVING STRONG CONTROL OF THE FAMILY‐WISE ERROR RATE

### 3.1 Closed testing procedures

Control of the type I error rate in a confirmatory clinical trial is paramount^11^ and, with two null hypotheses under consideration, the testing procedure should provide strong control of the FWER at the prespecified level α.^1^ Thus, we require

$$P_{\theta }(\text{Reject at least one true null hypothesis})\leq \alpha \;\text{for all}\;\theta .$$

We shall follow the general approach presented by Bretz et al,^12^ Schmidli et al^13^ and Jennison and Turnbull^14^ who ensure strong control of the FWER by constructing a closed testing procedure^15^ in which combination tests are carried out on the individual hypotheses. In addition to the null hypotheses *H*_01_: $\theta_{1}\leq 0$ and *H*_03_: $\theta_{3}\leq 0$, the closed testing procedure also considers the intersection hypothesis *H*_0, 13_ = *H*_01_ ∩ *H*_03_ which states that $\theta_{1}\leq 0$ and $\theta_{3}\leq 0$. We specify level α tests of *H*_01_, *H*_03_, and *H*_0, 13_. Then, *H*_01_ is rejected in the overall procedure if the individual level α tests reject *H*_01_ and *H*_0, 13_. Similarly, *H*_03_ is rejected overall if the individual level α tests reject *H*_03_ and *H*_0, 13_. For an explanation of why such a procedure protects the FWER and why all procedures that provide strong control of FWER can be interpreted as closed testing procedures, see Appendix A.

We refer to the periods of an adaptive enrichment design before and after the interim analysis as stages 1 and 2. In our closed testing procedure, we need a method for combining test statistics for hypotheses *H*_01_ and *H*_03_ to test the intersection hypothesis *H*_0, 13_ and a method to combine data across stages, bearing in mind that the decision about which subpopulations to recruit from in stage 2 depends on the stage 1 data. We describe these methods in the following sections.

### 3.2 Simes' test for the intersection hypothesis

Let $P_{1}^{\left(1\right)}$ and $P_{3}^{\left(1\right)}$ be *P*‐values for testing *H*_01_ and *H*_03_ based on stage 1 data. Then $P_{1}^{\left(1\right)}∼\text{Unif}(0,1)$ if $\theta_{1}=0$ and $P_{1}^{\left(1\right)}$ is stochastically larger than a Unif(0, 1) random variable if $\theta_{1}<0$; similarly, $P_{3}^{\left(1\right)}∼\text{Unif}(0,1)$ if $\theta_{3}=0$ and $P_{3}^{\left(1\right)}$ is stochastically larger than this if $\theta_{3}<0$. We can use Simes' method^2^ to create a *P*‐value for the intersection hypothesis *H*_0, 13_,

(2) $$P_{13}^{\left(1\right)}=\min\{2\min(P_{1}^{\left(1\right)},P_{3}^{\left(1\right)}),\;\max(P_{1}^{\left(1\right)},P_{3}^{\left(1\right)})\}.$$

Since $P_{1}^{\left(1\right)}$ and $P_{3}^{\left(1\right)}$ are based on nested groups of patients, these p‐values are positively associated and the results of Sarkar and Chang^16^ imply that Simes' test gives a valid (but conservative) *P*‐value for testing *H*_0, 13_.

If enrichment does not take place and stage 2 continues with recruitment from the full population, we define $P_{1}^{\left(2\right)}$ and $P_{3}^{\left(2\right)}$ to be p‐values for testing *H*_01_ and *H*_03_ based on data from stage 2 patients alone. Then, just as for stage 1 data, we construct the Simes p‐value

(3) $$P_{13}^{\left(2\right)}=\min\{2\min(P_{1}^{\left(2\right)},P_{3}^{\left(2\right)}),\;\max(P_{1}^{\left(2\right)},P_{3}^{\left(2\right)})\},$$

for testing the intersection hypothesis *H*_0, 13_.

If enrichment does take place, only patients from ${𝒮}_{1}$ are observed in stage 2 and we define the *P*‐value $P_{1}^{\left(2\right)}$ for *H*_01_ based on these observations. We cannot define a *P*‐value $P_{3}^{\left(2\right)}$ but this is not a problem as we no longer plan to test *H*_03_. In this case we set

(4) $$P_{13}^{\left(2\right)}=P_{1}^{\left(2\right)},$$

noting that *H*_0, 13_ implies $\theta_{1}\leq 0$ and hence $P_{13}^{\left(2\right)}=P_{1}^{\left(2\right)}$ is Unif(0, 1), or stochastically larger than this, under *H*_0,13_.

### 3.3 The weighted inverse normal combination test

In constructing level α tests of *H*_01_, *H*_03_, and *H*_0,13_, we need to combine *P*‐values from the two stages. In each case, we do this using a weighted inverse normal combination test.^3^, ^4^, ^5^

Consider first the level α test of *H*_01_. The stage 1 data give

$$Z_{1}^{\left(1\right)}={\hat{\theta }}_{1}^{\left(1\right)}\surd \{\lambda \tau \tilde{ℐ}\}∼N(\theta_{1}\surd \{\lambda \tau \tilde{ℐ}\},1),$$

and the associated *P*‐value is $P_{1}^{\left(1\right)}=1-\Phi (Z_{1}^{\left(1\right)})$ where Φ denotes the cumulative distribution function of a standard normal random variable. If the trial recruits from the full population in stage 2, we have

$$Z_{1}^{\left(2\right)}={\hat{\theta }}_{1}^{\left(2\right)}\surd \{\lambda (1-\tau )\tilde{ℐ}\}∼N(\theta_{1}\surd \{\lambda (1-\tau )\tilde{ℐ}\},1),$$

while, if enrichment occurs, we have

$$Z_{1}^{\left(2\right)}={\hat{\theta }}_{1}^{\left(2\right)}\surd \{(1-\tau )\tilde{ℐ}\}∼N(\theta_{1}\surd \{(1-\tau )\tilde{ℐ}\},1),$$

and in either case the associated *P*‐value is $P_{1}^{\left(2\right)}=1-\Phi (Z_{1}^{\left(2\right)})$.

Suppose $\theta_{1}=0$. Then, $Z_{1}^{\left(1\right)}∼N(0,1)$ and $P_{1}^{\left(1\right)}∼\text{Unif}(0,1)$. Conditional on the first stage data, $Z_{1}^{\left(2\right)}∼N(0,1)$ and $P_{1}^{\left(2\right)}∼\text{Unif}(0,1)$. Since the conditional distribution of $Z_{1}^{\left(2\right)}$ does not depend on the stage 1 data, we conclude that $Z_{1}^{\left(1\right)}$ and $Z_{1}^{\left(2\right)}$ are independent *N*(0, 1) random variables. Using pre‐specified weights *w*_1_ and *w*_2_ for which $w_{1}^{2}+w_{2}^{2}=1$, we define the combination test statistic

$$Z_{1}^{\left(c\right)}=w_{1}Z_{1}^{\left(1\right)}+w_{2}Z_{1}^{\left(2\right)},$$

and note that $Z_{1}^{\left(c\right)}∼N(0,1)$ when $\theta_{1}=0$.

Suppose now that $\theta_{1}<0$. We can write

$$Z_{1}^{\left(1\right)}=\theta_{1}\surd \{\lambda \tau \tilde{ℐ}\}+\epsilon_{1}^{\left(1\right)},$$

where $\epsilon_{1}^{\left(1\right)}∼N(0,1)$ and

$$Z_{1}^{\left(2\right)}=\theta_{1}c_{1}+\epsilon_{1}^{\left(2\right)},$$

where $\epsilon_{1}^{\left(2\right)}∼N(0,1)$, $\epsilon_{1}^{\left(2\right)}$ is independent of $\epsilon_{1}^{\left(1\right)}$, $c_{1}=\surd \{\lambda (1-\tau )\tilde{ℐ}\}$ if enrichment does not occur in stage 2 and $c_{1}=\surd \{(1-\tau )\tilde{ℐ}\}$ if enrichment does occur. Since

$$w_{1}\epsilon_{1}^{\left(1\right)}+w_{2}\epsilon_{1}^{\left(2\right)}∼N(0,1),$$

$Z_{1}^{\left(1\right)}<\epsilon_{1}^{\left(1\right)}$ and $Z_{1}^{\left(2\right)}<\epsilon_{1}^{\left(2\right)}$, it follows that $Z_{1}^{\left(c\right)}=w_{1}Z_{1}^{\left(1\right)}+w_{2}Z_{1}^{\left(2\right)}$ is stochastically smaller than a *N*(0, 1) random variable. Hence the test that rejects *H*_01_ if $Z_{1}^{\left(c\right)}>\Phi^{-1}(1-\alpha )$ has type I error rate less than or equal to α whenever $\theta_{1}\leq 0$, as required.

We construct a level α test of *H*_03_ in a similar way to that of *H*_01_. We have

$$Z_{3}^{\left(1\right)}={\hat{\theta }}_{3}^{\left(1\right)}\surd \{\tau \tilde{ℐ}\}∼N(\theta_{3}\surd \{\tau \tilde{ℐ}\},1),$$

from stage 1 data and, if enrichment does not occur, we have

$$Z_{3}^{\left(2\right)}={\hat{\theta }}_{3}^{\left(2\right)}\surd \{(1-\tau )\tilde{ℐ}\}∼N(\theta_{3}\surd \{(1-\tau )\tilde{ℐ}\},1),$$

from stage 2 data. In the case of no enrichment, we create the combination test statistic

$$Z_{3}^{\left(c\right)}=w_{1}Z_{3}^{\left(1\right)}+w_{2}Z_{3}^{\left(2\right)},$$

and we reject *H*_03_ if $Z_{3}^{\left(c\right)}>\Phi^{-1}(1-\alpha )$. The proof that this test controls the type I error rate follows the same lines as that for the test of *H*_01_ but, since we do not test *H*_03_ at all when enrichment occurs, this test is conservative even if $\theta_{3}=0$.

The level α test of the intersection hypothesis *H*_0, 13_ is constructed from the *P*‐values $P_{13}^{\left(1\right)}$ and $P_{13}^{\left(2\right)}$ as defined in Equations (2), (3) and (4). Under *H*_0, 13_, the positive correlation between ${\hat{\theta }}_{1}^{\left(1\right)}$ and ${\hat{\theta }}_{3}^{\left(1\right)}$ implies that $P_{13}^{\left(1\right)}$ is stochastically larger than a Unif(0, 1) random variable, even when $\theta_{1}=\theta_{3}=0$. Thus, $Z_{13}^{\left(1\right)}=\Phi^{-1}(1-P_{13}^{\left(1\right)})$ is stochastically smaller than a *N*(0, 1) random variable and we can write

(5) $$Z_{13}^{\left(1\right)}=\epsilon_{13}^{\left(1\right)}-\delta_{1},$$

where $\epsilon_{13}^{\left(1\right)}∼N(0,1)$ and $\delta_{1}$ is a positive random variable, not necessarily independent of $\epsilon_{13}^{\left(1\right)}$. If no enrichment occurs, by similar reasoning, the conditional distribution under *H*_0, 13_ of $Z_{13}^{\left(2\right)}=\Phi^{-1}(1-P_{13}^{\left(2\right)})$, given stage 1 data, is stochastically smaller than a *N*(0, 1) random variable. If enrichment does occur, $Z_{13}^{\left(2\right)}=Z_{1}^{\left(2\right)}$ and has conditional distribution $N(\theta_{1}\surd \{(1-\tau )\tilde{ℐ}\},1)$ given stage 1 data. It follows that, under *H*_0, 13_, we can write

(6) $$Z_{13}^{\left(2\right)}=\epsilon_{13}^{\left(2\right)}-\delta_{2},$$

where $\epsilon_{13}^{\left(2\right)}∼N(0,1)$ is independent of $\epsilon_{13}^{\left(1\right)}$ and $\delta_{2}$ is a positive random variable that may depend on $\epsilon_{13}^{\left(1\right)}$ and $\epsilon_{13}^{\left(2\right)}$. It follows from Equations (5) and (6) that, under *H*_0, 13_,

$$Z_{13}^{\left(c\right)}=w_{1}Z_{13}^{\left(1\right)}+w_{2}Z_{13}^{\left(2\right)}$$

is stochastically smaller than a *N*(0, 1) variable. Hence, the test that rejects *H*_0, 13_ if $Z_{13}^{\left(c\right)}>\Phi^{-1}(1-\alpha )$ has type I error rate less than or equal to α whenever $\theta_{1}\leq 0$ and $\theta_{3}\leq 0$.

### 3.4 Summary of the overall testing procedure

Let

$$S(P_{1},P_{2})=\min\{2\min(P_{1},P_{2}),\;\max(P_{1},P_{2})\},$$

be the function that converts *P*_1_ and *P*_2_ into a Simes *P*‐value and and define

(7) $$W(P^{\left(1\right)},P^{\left(2\right)})=1-\Phi \{w_{1}\Phi^{-1}(1-P^{\left(1\right)})+w_{2}\Phi^{-1}(1-P^{\left(2\right)})\},$$

the function that gives the *P*‐value when a weighted inverse normal combination test with weights *w*_1_ and *w*_2_ is applied to stage 1 and 2 *P*‐values *P*^(1)^ and *P*^(2)^. With this notation, Table 1 presents a summary of the closed testing procedure described above.

**TABLE 1:** Formulae for *P*‐values used to create level α tests of *H*_01_, *H*_03_, and *H*_0, 13_

	With no enrichment		
	*H*_01_	*H*_03_	*H*_0, 13_
Stage 1	$P_{1}^{\left(1\right)}=1-\Phi (Z_{1}^{\left(1\right)})$	$P_{3}^{\left(1\right)}=1-\Phi (Z_{3}^{\left(1\right)})$	$P_{13}^{\left(1\right)}=S(P_{1}^{\left(1\right)},P_{3}^{\left(1\right)})$
Stage 2	$P_{1}^{\left(2\right)}=1-\Phi (Z_{1}^{\left(2\right)})$	$P_{3}^{\left(2\right)}=1-\Phi (Z_{3}^{\left(2\right)})$	$P_{13}^{\left(2\right)}=S(P_{1}^{\left(2\right)},P_{3}^{\left(2\right)})$
Combined	$P_{1}^{\left(c\right)}=W(P_{1}^{\left(1\right)},P_{1}^{\left(2\right)})$	$P_{3}^{\left(c\right)}=W(P_{3}^{\left(1\right)},P_{3}^{\left(2\right)})$	$P_{13}^{\left(c\right)}=W(P_{13}^{\left(1\right)},P_{13}^{\left(2\right)})$
	**With enrichment**		
	***H*_01_**	***H*_03_**	***H*_0, 13_**
Stage 1	$P_{1}^{\left(1\right)}=1-\Phi (Z_{1}^{\left(1\right)})$	$P_{3}^{\left(1\right)}=1-\Phi (Z_{3}^{\left(1\right)})$	$P_{13}^{\left(1\right)}=S(P_{1}^{\left(1\right)},P_{3}^{\left(1\right)})$
Stage 2	$P_{1}^{\left(2\right)}=1-\Phi (Z_{1}^{\left(2\right)})$	—	$P_{13}^{\left(2\right)}=P_{1}^{\left(2\right)}$
Combined	$P_{1}^{\left(c\right)}=W(P_{1}^{\left(1\right)},P_{1}^{\left(2\right)})$	—	$P_{13}^{\left(c\right)}=W(P_{13}^{\left(1\right)},P_{13}^{\left(2\right)})$

In a trial where enrichment does not occur and patients are recruited from the full population in stage 2, we reject *H*_01_ overall if $P_{1}^{\left(c\right)}\leq \alpha$ and $P_{13}^{\left(c\right)}\leq \alpha$, and we reject *H*_03_ overall if $P_{3}^{\left(c\right)}\leq \alpha$ and $P_{13}^{\left(c\right)}\leq \alpha$. If enrichment occurs, *H*_01_ is rejected overall if $P_{1}^{\left(c\right)}\leq \alpha$ and $P_{13}^{\left(c\right)}\leq \alpha$ but it is not possible to test *H*_03_ as there is no $P_{3}^{\left(2\right)}$ to use in the combination test of *H*_03_; this is in keeping with the decision to enrich which implies it is no longer desired to test *H*_03_.

## 4 OPTIMIZING AN ADAPTIVE ENRICHMENT DESIGN

### 4.1 Bayesian decision framework

An enrichment design, as described in Section 2.2, that applies the closed testing procedure presented in Section 3 will protect the FWER regardless of the decision rule that determines when to enrich in stage 2. This gives us the opportunity to apply Bayesian decision theory^17^ to optimize the enrichment decision rule for our chosen criterion. This decision theoretic approach requires the specification of a prior distribution for θ and a gain, or utility, function that assigns a value to the final outcome of the study.

*The decision rule*. We denote the sufficient statistic for $\theta =(\theta_{1},\theta_{2})$ based on stage 1 data by $X_{1}=({\hat{\theta }}_{1}^{\left(1\right)},{\hat{\theta }}_{2}^{\left(1\right)})$. Note that $\left(\theta_{1},\theta_{2}\right)$ determines $\left(\theta_{1},\theta_{3}\right)$ and vice versa, so *X*_1_ is also the sufficient statistic for $\left(\theta_{1},\theta_{3}\right)$. We shall consider decision rules that are functions of *X*_1_. The decision under rule *d* is specified through the function *d*(*X*_1_) taking values in {1, 2}, with

$$\begin{array}{ll} d(X_{1})=1 & \Rightarrow \text{Enrich in stage 2,}\\ d(X_{1})=2 & \Rightarrow \text{Do not enrich in stage 2.} \end{array}$$

The form of the sufficient statistic *X*_2_ for θ based on stage 2 data depends on which decision is taken. If *d*(*X*_1_) = 1, enrichment occurs and $X_{2}={\hat{\theta }}_{1}^{\left(2\right)}$, while if *d*(*X*_1_) = 2 enrichment does not occur and $X_{2}=({\hat{\theta }}_{1}^{\left(2\right)},{\hat{\theta }}_{2}^{\left(2\right)})$. In either case we write *X* = (*X*_1_, *d*(*X*_1_), *X*_2_) to summarize the full set of data at the end of the study and the decision taken at the interim analysis.

*The prior distribution for* θ. We assume a continuous prior distribution for $\theta =(\theta_{1},\theta_{2})$ is specified and we denote the probability density function of the prior distribution by π(θ).

*The gain function*. The gain function G(θ,X) denotes the value assigned to the outcome of the study when θ is the parameter vector and we observe *X* = (*X*_1_, *d*(*X*_1_), *X*_2_). Note that we can deduce from *X* which of the hypotheses *H*_01_ and *H*_03_ are rejected in the final analysis.

Let ${ℛ}_{1}$ be the indicator variable of the event that *H*_01_ is rejected but *H*_03_ is not rejected, and let ${ℛ}_{3}$ be the indicator variable of the event that *H*_03_ is rejected. Both ${ℛ}_{1}$ and ${ℛ}_{3}$ are functions of *X*. In this paper we shall consider the gain function

(8) $$G(\theta ,X)=\lambda \theta_{1}{ℛ}_{1}+\theta_{3}{ℛ}_{3}.$$

Here, the gain is deemed to be proportional to the size of the population for which a treatment effect is found and also to the average treatment effect for patients in that population.

Other forms of gain function are possible: the key feature is that they are constructed based on the possible outcomes of the trial. A general form of gain function should capture the importance of each of these possible outcomes, for example, if we define $\gamma_{1}(\theta ,X)$ to represent the benefit of rejecting *H*_01_ and $\gamma_{3}(\theta ,X)$ to represent the benefit of rejecting *H*_03_, then the gain function will be

$$G(\theta ,X)=\gamma_{1}(\theta ,X){ℛ}_{1}+\gamma_{3}(\theta ,X){ℛ}_{3}.$$

The choice of $\gamma_{1}(\theta ,X)$ and $\gamma_{3}(\theta ,X)$ may reflect both the treatment effect as seen in Equation (8) and the estimates of $\theta_{1}$ and $\theta_{3}$ which can be constructed from *X*. In our formulation of the design question, the total sample size is fixed, so we have not included a cost of treating patients in the study in the overall gain function: such a cost would be required if we were to include the option of stopping for futility at the interim analysis. One could also consider adding other important outcomes from the trial such as the safety profile of the treatment. The application of the methods that follow is not particularly dependent on the choice of gain function, although the choice of gain function will influence what is optimal.

### 4.2 Computing the Bayes optimal design

With the prior distribution π and gain function *G* specified, we wish to find the decision rule *d* that maximises the Bayes expected gain of the trial E{G(θ,X)}, where the expectation is over both the prior distribution for θ and the distribution of *X* given θ.

We denote the conditional density function of *X*_1_ given θ by $f_{X_{1}|\theta }(x_{1}|\theta )$, the density of the marginal distribution of *X*_1_ by $f_{X_{1}}(x_{1})$, and the conditional density of *X*_2_ given θ and decision *d*(*x*_1_) by $f_{X_{2}|\theta ,d}(x_{2}|\theta ,d(x_{1}))$. Let $\pi_{\theta |X_{1}}(\theta |x_{1})$ be the density of the posterior distribution of θ given *X*_1_ = *x*_1_, so

$$\pi (\theta )f_{X_{1}|\theta }(x_{1}|\theta )=f_{X_{1}}(x_{1})\;\pi_{\theta |X_{1}}(\theta |x_{1}).$$

Then the expected gain when applying decision rule *d* is

(9) $$\begin{array}{ll} E\{G(\theta ,X)\} & =\int_{\theta }\int_{x_{1}}\int_{x_{2}}\pi (\theta )f_{X_{1}|\theta }(x_{1}|\theta )f_{X_{2}|\theta ,d}(x_{2}|\theta ,d(x_{1}))\;G(\theta ,(x_{1},d(x_{1}),x_{2}))\;dx_{2}\;dx_{1}\;d\theta \\ & =\int_{x_{1}}f_{X_{1}}(x_{1})\int_{\theta }\int_{x_{2}}\pi_{\theta |X_{1}}(\theta |x_{1})f_{X_{2}|\theta ,d}(x_{2}|\theta ,d(x_{1}))\;G(\theta ,(x_{1},d(x_{1}),x_{2}))\;dx_{2}\;d\theta \;dx_{1}. \end{array}$$

It is evident from (9) that the optimal decision rule can be found by choosing *d*(*x*_1_) to maximize

(10) $$\int_{\theta }\int_{x_{2}}\pi_{\theta |X_{1}}(\theta |x_{1})f_{X_{2}|\theta ,d}(x_{2}|\theta ,d(x_{1}))\;G(\theta ,(x_{1},d(x_{1}),x_{2}))\;dx_{2}\;d\theta =E\{G(\theta ,X)\;|\;X_{1}=x_{1},d(x_{1})\},$$

for each *x*_1_. That is, we choose the enrichment decision that maximizes the conditional expected gain given the stage 1 data under the posterior distribution of θ at the interim analysis.

Given observed stage 1 data $X_{1}=x_{1}=({\hat{\theta }}_{1}^{\left(1\right)},{\hat{\theta }}_{2}^{\left(1\right)})$, we need to compare values of the integral (10) in the two cases *d*(*x*_1_) = 1 (enrichment) and *d*(*x*_1_) = 2 (no enrichment). Since this integral is not analytically tractable, we evaluate it by Monte Carlo simulation. To do this, we draw a sample $\left\{\theta_{i}=(\theta_{i,1},\theta_{i,2}\right)$, *i* = 1, … , *M*}, from the posterior distribution $\pi_{\theta |X_{1}}(\theta |x_{1})$ and find the conditional expected gain under each $\theta_{i}$ for the two options, “enrich” and “do not enrich.” We take the average gain over this sample of $\theta_{i}$ values as our estimate of the conditional expected gain for each option. We conclude that the decision *d*(*x*_1_) giving the larger of the two values for the conditional expected gain is the Bayes optimal decision when $X_{1}=x_{1}=({\hat{\theta }}_{1}^{\left(1\right)},{\hat{\theta }}_{2}^{\left(1\right)})$.

In assessing the decision to enrich, *d*(*x*_1_) = 1, when $X_{1}=x_{1}=({\hat{\theta }}_{1}^{\left(1\right)},{\hat{\theta }}_{2}^{\left(1\right)})$ we apply the definitions of Section 3 to find the critical value $\kappa (x_{1})$ such that ${\hat{\theta }}_{1}^{\left(2\right)}\geq \kappa (x_{1})$ implies $P_{1}^{\left(c\right)}\leq \alpha$ and $P_{13}^{\left(c\right)}\leq \alpha$, so *H*_01_ is rejected in the closed testing procedure. We compute $P({\hat{\theta }}_{1}^{\left(2\right)}\geq \kappa (x_{1})\;|\;\theta_{1}=\theta_{i,1},{\hat{\theta }}_{1}^{\left(1\right)},d(x_{1})=1)$ for each *i* = 1, … , *M* and combine the results to obtain the estimate of the conditional expected gain

(11) $$\hat{E}\{G(\theta ,X)\;|\;X_{1}=x_{1}=({\hat{\theta }}_{1}^{\left(1\right)},{\hat{\theta }}_{2}^{\left(1\right)}),d(x_{1})=1\}=\frac{1}{M}\overset{M}{\underset{i=1}{\sum }}\lambda \theta_{i,1}P({\hat{\theta }}_{1}^{\left(2\right)}\geq \kappa (x_{1})\;|\;\theta_{1}=\theta_{i,1},{\hat{\theta }}_{1}^{\left(1\right)},d(x_{1})=1).$$

If *d*(*x*_1_) = 2 and the trial continues without enrichment, the possibilities in stage 2 are more complex. In this case, for each *i* = 1, … , *M* we continue to simulate the remainder of the trial by generating $\left({\hat{\theta }}_{i,1}^{\left(2\right)},{\hat{\theta }}_{i,2}^{\left(2\right)}\right)$ under $\theta =\theta_{i}$ and evaluating the gain (8) with $\theta =\theta_{i}$ and $x=(({\hat{\theta }}_{1}^{\left(1\right)},{\hat{\theta }}_{2}^{\left(1\right)}),2,({\hat{\theta }}_{i,1}^{\left(2\right)},{\hat{\theta }}_{i,2}^{\left(2\right)}))$. Combining these results gives the estimate of the conditional expected gain

(12) $$\hat{E}\{G(\theta ,X)\;|\;X_{1}=x_{1}=({\hat{\theta }}_{1}^{\left(1\right)},{\hat{\theta }}_{2}^{\left(1\right)}),d(x_{1})=2\}=\frac{1}{M}\overset{M}{\underset{i=1}{\sum }}G(\theta_{i},(({\hat{\theta }}_{1}^{\left(1\right)},{\hat{\theta }}_{2}^{\left(1\right)}),2,({\hat{\theta }}_{i,1}^{\left(2\right)},{\hat{\theta }}_{i,2}^{\left(2\right)}))).$$

The value of *M* used in these simulations should be chosen to give the desired level of accuracy. We have found *M* = 10^5^ or 10^6^ to give sufficient accuracy in the examples we have studied.

### 4.3 Determining the decision rule and decision boundary

In order to find the operating characteristics of a proposed adaptive enrichment design we must be able to repeatedly simulate the design in full. This requires repeated application of the interim decision rule that specifies the optimal design for a given prior π and gain function *G*: thus we need to know the optimal decision for all possible values of $x_{1}=({\hat{\theta }}_{1}^{\left(1\right)},{\hat{\theta }}_{2}^{\left(1\right)})$. We present an algorithm that enables the computation of the optimal decision rule over a large square region, *A*, such that *P*(*X*_1_ ∈ *A*) is very close to 1. The algorithm divides this region into an array of much smaller squares and determines the optimal decision for values of *x*_1_ in each small square. With simple extrapolation beyond the boundaries of *A*, this process divides the plane into two regions, *A*_*E*_ where the optimal decision is to enrich, and *A*_*C*_ where it is optimal to continue recruitment in the full population.

Experience shows that the two regions *A*_*E*_ and *A*_*C*_ are quite regular in shape and this fact allows us to reduce the computation needed to find the optimal decision rule. We first divide *A* into four subsquares and determine the optimal decisions at the vertices of these squares. Then, if the same decision is optimal at all four vertices we record this as the optimal decision for all points in that square. If, however, both decisions are optimal for at least one vertex we subdivide this square into four smaller squares. In the next iterative step, we consider the set of squares of the smallest size and for each of these we either record an optimal decision for the whole square or subdivide the square into four smaller ones. We continue this iterative process until we reach squares of the desired size. Further details of this method and a discussion of its accuracy are given in Appendix B. The results of these calculations are 2‐fold. First, the list of optimal decisions for each small square provides the information needed to implement the optimal adaptive decision rule. Secondly, the results can be presented graphically to help visualize the optimal decision rule.

### 4.4 Assessing the performance of an optimized trial design

Suppose the decision rule of an optimized adaptive enrichment design is defined by regions *A*_*E*_ and *A*_*C*_ as described above. We assess the overall performance of this design by simulation. For each replicate *i* = 1, … , *N*, we generate a parameter vector $\theta_{i}=(\theta_{i,1},\theta_{i,2})$ then simulate stage 1 data $x_{i,1}=({\hat{\theta }}_{i,1}^{\left(1\right)},{\hat{\theta }}_{i,2}^{\left(1\right)})$ assuming $\theta =\theta_{i}$. We determine whether *x*_*i*, 1_ is in *A*_*E*_ or *A*_*C*_, set *d*(*x*_*i*, 1_) = 1 or 2 accordingly, and apply this decision, still assuming $\theta =\theta_{i}$, as we generate the stage 2 data: $x_{i,2}={\hat{\theta }}_{i,1}^{\left(2\right)}$ if *d*(*x*_*i*, 1_) = 1 (enrichment), or $x_{i,2}=({\hat{\theta }}_{i,1}^{\left(2\right)},{\hat{\theta }}_{i,2}^{\left(2\right)})$ if *d*(*x*_*i*, 1_) = 2 (no enrichment). Finally, we determine which hypotheses are rejected and evaluate the gain function for these outcomes when $\theta =\theta_{i}$. Averaging over the *N* replicates gives the estimate

$$\hat{E}\{G(\theta ,X)\}=\frac{1}{N}\overset{N}{\underset{i=1}{\sum }}G(\theta_{i},(x_{i,1},d(x_{i,1}),x_{i,2})).$$

The same set of simulated data can be used to estimate other properties of the design such as the probabilities of rejecting each null hypothesis. In our simulations we have used *N* = 10^6^, so sampling error for the estimates reported is negligible.

One might ask whether it would be helpful to generate multiple replicates of the stage 2 data for each $\theta_{i}$ and *x*_1, *i*_. However, the distribution of $\theta_{i}$ and *x*_1, *i*_ accounts for much of the variability of G(θ,X) and it is more efficient to use the available computational effort to increase the number of replicates, *N*, of the first stage data. Of course, this approach relies on our having carried out initial work to find the regions *A*_*E*_ and *A*_*C*_ that define the optimal decision rule, and in doing this we will have generated multiple samples of stage 2 data conditional on particular values of *X*_1_.

## 5 TWO NONADAPTIVE DESIGNS

There are two further options that should be considered when an adaptive enrichment design is envisaged. The first is a design in which patients are recruited from the full population throughout the trial, but both null hypotheses *H*_01_ and *H*_03_ are tested at the end. We shall refer to this as the Fixed Full population (FF) design. The other possibility is a Fixed Subpopulation (FS) design, in which subjects are only recruited from the subpopulation and only the hypothesis *H*_01_ is tested.

*The Fixed Full population design*. For comparability with other designs, we assume the same total sample size, *n*, as in Section 2.2. Thus, λn patients are recruited from ${𝒮}_{1}$ and (1−λ)n from ${𝒮}_{2}$. With $\tilde{ℐ}$ as defined in (1), the data provide estimates

$${\hat{\theta }}_{1}∼N(\theta_{1},{\left(\lambda \tilde{ℐ}\right)}^{-1})),$$

and

$${\hat{\theta }}_{3}∼N(\theta_{3},{\left(\tilde{ℐ}\right)}^{-1}),$$

and the joint distribution of $\left({\hat{\theta }}_{1},{\hat{\theta }}_{3}\right)$ is bivariate normal with correlation $\sqrt{\lambda }$.

The *P*‐values for testing *H*_01_ and *H*_03_ are

$$P_{1}=1-\Phi ({\hat{\theta }}_{1}\surd \{\lambda \tilde{ℐ}\})\;\text{and}\;P_{3}=1-\Phi ({\hat{\theta }}_{3}\surd \tilde{ℐ}),$$

respectively, and Simes' method gives the p‐value

$$P_{13}=\min\{2\;\min(P_{1},P_{3}),\;\max(P_{1},P_{3})\}$$

for the intersection hypothesis *H*_0, 13_. Applying the closed testing procedure, we reject *H*_01_ overall if $P_{1}\leq \alpha$ and $P_{13}\leq \alpha$, and we reject *H*_03_ overall if $P_{3}\leq \alpha$ and $P_{13}\leq \alpha$.

There are reasons why the FF design may be more efficient than the optimal adaptive design if the prior π(θ) is concentrated on values of θ under which enrichment is unlikely to occur. Suppose an adaptive design is conducted and enrichment does not occur. With suitable weights in the combination rule (7), the adaptive design's *P*‐values $P_{1}^{\left(c\right)}$ and $P_{3}^{\left(c\right)}$, as shown in Table 1, are equal to the *P*_1_ and *P*_3_ obtained when the same data are observed in the FF design. However, $P_{13}^{\left(c\right)}=W(P_{13}^{\left(1\right)},P_{13}^{\left(2\right)})$ differs from the *P*_13_ arising from the same data in the FF design. Since *P*_13_ in the FF design is based on the sufficient statistics for $\theta_{1}$ and $\theta_{3}$ in the full data set, it provides a more powerful test of *H*_0, 13_ than the adaptive design's $P_{13}^{\left(c\right)}$. The requirement to use $P_{13}^{\left(c\right)}$ rather than *P*_13_ to test *H*_0, 13_ is the price we pay for the adaptive design's flexibility to enrich on other occasions: if such occasions are not particularly likely under the prior π(θ), it is plausible that the FF design will be superior.

*The Fixed Subpopulation design*. In the FS design, all *n* subjects are recruited from ${𝒮}_{1}$. These provide the estimate

$${\hat{\theta }}_{1}∼N(\theta_{1},{\tilde{ℐ}}^{-1})),$$

and the *P*‐value

$$P_{1}=1-\Phi ({\hat{\theta }}_{1}\surd \tilde{ℐ}),$$

and *H*_01_ is rejected if $P_{1}\leq \alpha$. In this design *H*_03_ is not tested.

We can expect the FS design to perform well when the prior π(θ) is such that the optimal adaptive design is highly likely to enrich. Then, the FS design has the benefit of a larger sample size from ${𝒮}_{1}$ and, hence, a more accurate estimate ${\hat{\theta }}_{1}$. Furthermore, the FS design only tests *H*_01_ and so does not have to make a multiplicity adjustment for testing two hypotheses.

## 6 EXAMPLES

### 6.1 One‐point prior distributions

We consider a Phase III clinical trial as described in Section 2.1 where the subpopulations ${𝒮}_{1}$ and ${𝒮}_{2}$ are of equal size, so λ=0.5. We set the FWER to be α=0.025 and suppose the total sample size *n* would provide power 0.9 to detect a treatment effect of size 10 when testing only the hypothesis *H*_03_ in a nonadaptive design. This leads to the total information

$$\tilde{ℐ}={\left(\frac{\Phi^{-1}(0.9)+\Phi^{-1}(0.975)}{10}\right)}^{2}=0.105,$$

which is, for example, the information provided by a total sample size *n* = 264 when patient responses have standard deviation σ=25. In adaptive enrichment designs we suppose the interim analysis occurs after half the total sample has been observed, thus τ=0.5. Then, with λ=0.5, τ=0.5 and $\tilde{ℐ}=0.105$, the interim estimates ${\hat{\theta }}_{1}^{\left(1\right)}$ and ${\hat{\theta }}_{1}^{\left(2\right)}$ have SD 6.15.

In order to gain insight into how adaptive designs function and what they may achieve, we first consider cases where the prior distribution for θ places probability mass 1 at a single point, $\theta =\theta_{0}=(\theta_{0,1},\theta_{0,2})$. For given $\theta_{0}$, we derived the decision rule for the adaptive enrichment (AE) design that maximises the expected gain, using the gain function G(θ,X) specified in (8). For comparison, we also computed properties under $\theta =\theta_{0}$ of the FF design, which recruits from the full population throughout the trial, and the FS design which only recruits from the subpopulation. Results presented in Table 2 for selected values of $\theta_{0}$ show each type of design, FF, FS, and AE, to be optimal for certain values of $\theta_{0}$.

**TABLE 2:** Properties of fixed subpopulation (FS), fixed full population (FF), and optimal adaptive enrichment (AE) designs when $\theta =\theta_{0}=(\theta_{0,1},\theta_{0,2})$. Here $P({ℛ}_{1})$ is the probability that only *H*_01_ is rejected and $P({ℛ}_{3})$ the probability that *H*_03_ is rejected. The AE design is optimized for the prior distribution with probability 1 at the single point $\theta =\theta_{0}$. In each case, the design with the highest expected gain is highlighted

$\theta_{0,1}$	$\theta_{0,2}$	$\theta_{0,3}$	Trial design	$P({ℛ}_{1})$	$P({ℛ}_{3})$	*P*(Enrich)	E{G(θ,X)}
10	2	6	**FS**	0.90	—	—	**4.50**
		FF	0.14	0.46	—	3.48
		AE	0.50	0.23	0.71	3.89
10	4	7	FS	0.90	—	—	4.50
		FF	0.08	0.58	—	4.46
		**AE**	0.25	0.46	0.38	**4.51**
10	6	8	FS	0.90	—	—	4.50
		**FF**	0.04	0.69	—	**5.68**
		AE	0.08	0.64	0.13	5.55
10	10	10	FS	0.90	—	—	4.50
		**FF**	0.01	0.86	—	**8.60**
		AE	0.01	0.83	0.00	8.34
12	2	7	**FS**	0.97	—	—	**5.84**
		FF	0.15	0.60	—	5.15
		AE	0.50	0.36	0.58	5.58
12	4	8	FS	0.97	—	—	5.84
		FF	0.09	0.71	—	6.20
		**AE**	0.25	0.60	0.28	**6.30**
12	6	9	FS	0.97	—	—	5.84
		**FF**	0.04	0.80	—	**7.44**
		AE	0.09	0.76	0.10	7.38
14	2	8	FS	1.00	—	—	6.97
		FF	0.15	0.73	—	6.83
		**AE**	0.40	0.54	0.39	**7.13**
14	4	9	FS	1.00	—	—	6.97
		FF	0.08	0.82	—	7.90
		**AE**	0.19	0.74	0.17	**7.97**
14	6	10	FS	1.00	—	—	6.97
		**FF**	0.04	0.88	—	**9.10**
		AE	0.07	0.86	0.06	9.07

**FIGURE 1:**
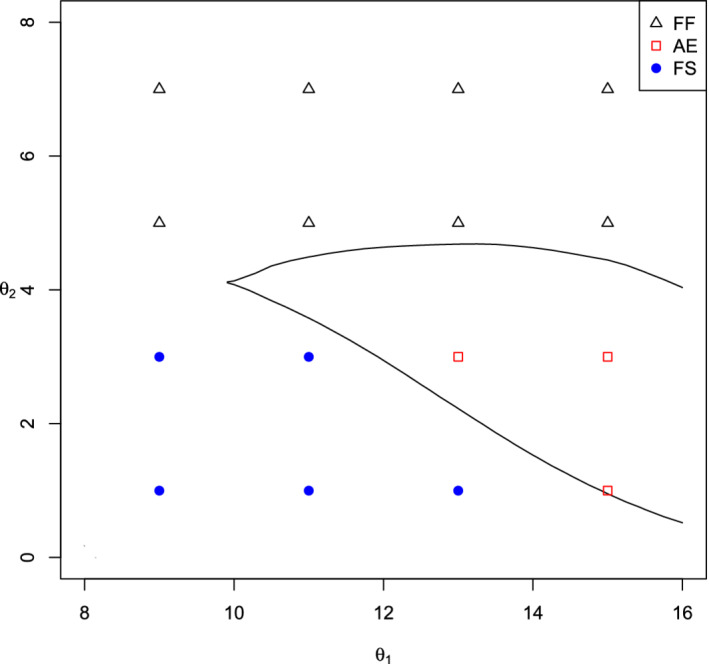
Regions of θ values in which each of the Fixed Full population (FF), Fixed Subpopulation (FS), and optimal Adaptive Enrichment (AE) designs give the highest value of E{G(θ,X)} [Color figure can be viewed at wileyonlinelibrary.com]

We carried out further calculations on a grid of values of $\theta_{0}$ to find the regions where each type of design is optimal. These regions are shown in Figure 1.

We note that the FF design is optimal when $\theta_{0,3}=0.5\;(\theta_{0,1}+\theta_{0,2})$ is large or $\theta_{0,1}$ is only a little larger than $\theta_{0,2}$. The FS design is optimal when $\theta_{0,1}$ is substantially larger than $\theta_{0,2}$ and $\theta_{0,2}$ is small. This leaves a region of $\theta_{0}$ values where the AE design is optimal, offering a modest increase in expected gain over both fixed designs. The advantage of the AE design over the FF design is largest in cases such as $\theta_{0}=(10,2)$ and $\theta_{0}=(12,2)$, where $\theta_{0,2}$ is small and the AE design has a high probability of enrichment and rejection of *H*_01_ only. Although the FS design has even higher expected gain in these cases, investigators may be reluctant to make such an early decision to ignore subpopulation ${𝒮}_{2}$ completely, in which case the key comparison is between AE and FF designs.

In extreme cases such as θ=(10,10) where both $\theta_{0,1}$ and $\theta_{0,2}$ are high, there is a high probability that the AE design does not enrich and so has the same final dataset as the FF design. As discussed in Section 5, the AE design uses a different form of $P_{13}^{\left(c\right)}$ and this leads to less efficient use of the final data when enrichment does not occur and a lower expected gain than for the FF design.

Since the AE design is optimized with knowledge of the value of $\theta_{0}$, its advantage when it is superior to both fixed designs does not stem from having improved estimates of the true treatment effects at the interim analysis. Rather, the decision to enrich or not is based on the likelihood that current data, summarized as $\left({\hat{\theta }}_{1}^{\left(1\right)},{\hat{\theta }}_{1}^{\left(2\right)}\right)$, will lead to eventual rejection of *H*_01_ or *H*_03_. This suggests that the AE design may have an even greater advantage in situations where the prior distribution for θ is more dispersed, since then it can also exploit the information about θ that becomes available at the interim analysis. We shall assess the performance of designs under dispersed prior distributions for θ in the next Section.

### 6.2 Proper prior distributions for θ

In practice, one expects there to be considerable uncertainty about the true treatment effect. We capture this uncertainty in a bivariate normal prior distribution for θ,

(13) $$\left(\begin{array}{c} \theta_{1}\\ \theta_{2} \end{array}\right)∼N_{2}\left(\left(\begin{array}{c} \mu_{1}\\ \mu_{2} \end{array}\right),\left(\begin{array}{cc} \sigma_{1}^{2} & \rho \;\sigma_{1}\sigma_{2}\\ \rho \;\sigma_{1}\sigma_{2} & \sigma_{2}^{2} \end{array}\right)\right).$$

**FIGURE 2:**
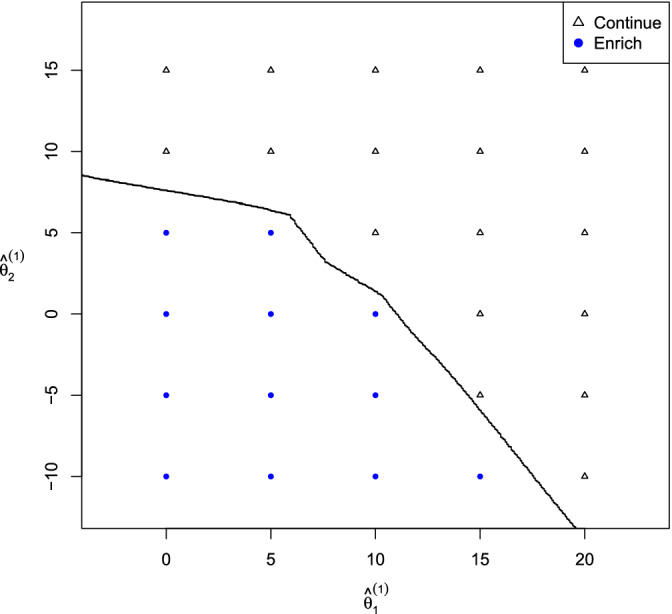
An example of a Bayes optimal decision rule for an adaptive enrichment trial [Color figure can be viewed at wileyonlinelibrary.com]

Figure 2 shows the enrichment decision rule for the Bayes optimal adaptive enrichment trial when $\mu_{1}=12$, $\mu_{2}=2$, $\sigma_{1}^{2}=\sigma_{2}^{2}=25$ and ρ=0.75. The sharp angles in the decision boundary arise from discontinuities in the way ${\hat{\theta }}_{1}^{\left(1\right)}$ and ${\hat{\theta }}_{1}^{\left(2\right)}$ determine $P_{1}^{\left(1\right)}$, $P_{3}^{\left(1\right)}$, and $P_{13}^{\left(1\right)}$ and how these *P*‐values appear in the criteria for the closed testing procedure to reject *H*_01_ or *H*_03_.

Enrichment occurs when there is a low conditional probability of rejecting *H*_03_, given the prior and current data. This includes cases where both ${\hat{\theta }}_{1}^{\left(1\right)}$ and ${\hat{\theta }}_{1}^{\left(2\right)}$ are low so rejection of *H*_01_ is also unlikely: one could add a rule to stop for futility in such cases. When ${\hat{\theta }}_{1}^{\left(1\right)}$ is high, so that rejection of *H*_01_ is very likely, the trial is not enriched, even for lower values of ${\hat{\theta }}_{2}^{\left(1\right)}$, as long as it is feasible that *H*_03_ will also be rejected.

Table 3 shows properties of the Bayes optimal AE design, along with properties of the nonadaptive FF and FS designs, for prior distributions centred at the values of $\theta_{0}$ considered in Table 2 but with $\sigma_{1}^{2}=\sigma_{2}^{2}=25$ and ρ=0.75. In contrast with the results of Table 2, the AE design has higher expected gain than the FS design in all these examples with a dispersed prior.

**TABLE 3:** Properties of fixed subpopulation (FS), fixed full population (FF), and optimal adaptive enrichment (AE) designs when θ has the prior distribution given by (13). Here $P({ℛ}_{1})$ is the probability that only *H*_01_ is rejected and $P({ℛ}_{3})$ the probability that *H*_03_ is rejected

$\mu_{1}$	$\mu_{2}$	$\sigma_{1}^{2}$	$\sigma_{2}^{2}$	ρ	Trial design	$P({ℛ}_{1})$	$P({ℛ}_{3})$	*P*(Enrich)	E{G(θ,X)}
10	2	25	25	0.75	FS	0.75	—	—	4.42
					FF	0.10	0.48	—	4.89
					**AE**	0.25	0.38	0.53	**4.98**
10	4	25	25	0.75	FS	0.75	—	—	4.42
					**FF**	0.06	0.54	—	**5.64**
					AE	0.15	0.48	0.37	5.63
10	6	25	25	0.75	FS	0.75	—	—	4.42
					**FF**	0.04	0.61	—	**6.52**
					AE	0.08	0.57	0.23	6.43
10	10	25	25	0.75	FS	0.75	—	—	4.43
					**FF**	0.01	0.72	—	**8.59**
					AE	0.01	0.70	0.02	8.43
12	2	25	25	0.75	FS	0.84	—	—	5.57
					FF	0.12	0.55	—	6.09
					**AE**	0.29	0.44	0.49	**6.23**
12	4	25	25	0.75	FS	0.84	—	—	5.57
					FF	0.08	0.62	—	6.86
					**AE**	0.18	0.55	0.33	**6.91**
12	6	25	25	0.75	FS	0.84	—	—	5.57
					**FF**	0.05	0.68	—	**7.77**
					AE	0.10	0.64	0.21	7.72
14	2	25	25	0.75	FS	0.91	—	—	6.72
					FF	0.14	0.63	—	7.33
					**AE**	0.32	0.50	0.44	**7.53**
14	4	25	25	0.75	FS	0.91	—	—	6.72
					FF	0.10	0.69	—	8.13
					**AE**	0.19	0.62	0.29	**8.21**
14	6	25	25	0.75	FS	0.91	—	—	6.72
					FF	0.06	0.74	—	9.03
					**AE**	0.11	0.71	0.18	**9.04**

The AE design has higher expected gain than the FF design in six of the ten examples — but the margin of superiority is not great. Thus, there is not much evidence that the enrichment design profits from information about θ at the interim analysis. The explanation for this is that, in the examples of Table 3, the posterior distribution of θ after seeing the interim data is still widely dispersed, with the SDs for $\theta_{1}$ and $\theta_{2}$ equal to 3.59. This is not just a feature of our particular examples. Suppose a study's total sample size is chosen so that a final test of *H*_03_: $\theta_{3}\leq 0$ with type I error rate 0.025 has power 0.9 when $\theta_{3}=\delta$. With no enrichment, the SD of the final ${\hat{\theta }}_{3}$ is 0.31δ. If there are two equally sized subpopulations, the interim estimates of $\theta_{1}$ and $\theta_{2}$ based on half of the total data have SD 0.62δ. The posterior variance of $\theta_{1}$ and $\theta_{2}$ at the interim analysis depends on the prior variances of $\theta_{1}$ and $\theta_{2}$ and, to a small degree, on the prior correlation. If, as in the examples of Table 3, the prior has $Var\;(\theta_{1})=Var\;(\theta_{2})={\left(\delta /2\right)}^{2}$, the posterior SDs of $\theta_{1}$ and $\theta_{2}$ at the interim analysis will be around 0.36δ and a credible interval for $\theta_{1}$ or $\theta_{2}$ could easily contain both 0 and δ. On the other hand, the lower prior variances $Var\;(\theta_{1})=Var\;(\theta_{2})={\left(\delta /4\right)}^{2}$ lead to posterior SDs around 0.23δ—only slightly lower than the prior SDs of 0.25δ. Thus, in cases where the prior variance is high, considerable uncertainty about $\theta_{1}$ and $\theta_{2}$ remains at the interim analysis, while if the prior variance is low, the interim data have little impact on the posterior distribution of $\theta_{1}$ and $\theta_{2}$.

Table 4 presents results for a further selection of prior distributions for θ. The examples show that the prior correlation, ρ, has a small effect on expected gain but very little effect on the relative performance of different designs.

**TABLE 4:** Properties of fixed subpopulation (FS), fixed full population (FF), and optimal adaptive enrichment (AE) designs when θ has the prior distribution given by (13)

Prior parameters					E{G(θ,X)}			*P*(Enrich)
$\mu_{1}$	$\mu_{2}$	$\sigma_{1}^{2}$	$\sigma_{2}^{2}$	ρ	FS	FF	AE	for AE
10	2	0	0	—	**4.50**	3.48	3.89	0.71
10	2	1	1	0	**4.47**	3.55	3.93	0.69
10	2	1	1	0.75	**4.47**	3.58	3.95	0.69
10	2	4	4	0	**4.42**	3.74	4.04	0.64
10	2	4	4	0.75	**4.42**	3.81	4.09	0.64
10	2	16	16	0	4.38	4.33	**4.50**	0.55
10	2	16	16	0.75	4.38	4.52	**4.65**	0.55
12	2	0	0	—	**5.84**	5.15	5.58	0.58
12	2	1	1	0	**5.81**	5.18	5.58	0.56
12	2	1	1	0.75	**5.81**	5.19	5.59	0.56
12	2	4	4	0	**5.74**	5.29	5.61	0.52
12	2	4	4	0.75	**5.74**	5.34	5.67	0.53
12	2	16	16	0	5.60	5.66	**5.86**	0.49
12	2	16	16	0.75	5.60	5.80	**5.99**	0.49
10	4	0	0	—	4.50	4.46	**4.51**	0.39
10	4	1	1	0	4.47	4.51	**4.56**	0.39
10	4	1	1	0.75	4.47	4.52	**4.57**	0.38
10	4	4	4	0	4.42	4.66	**4.68**	0.36
10	4	4	4	0.75	4.42	4.71	**4.75**	0.37
10	4	16	16	0	4.38	**5.14**	**5.14**	0.35
10	4	16	16	0.75	4.38	**5.31**	**5.31**	0.37
12	4	0	0	—	5.84	6.20	**6.30**	0.28
12	4	1	1	0	5.81	6.21	**6.31**	0.28
12	4	1	1	0.75	5.81	6.22	**6.32**	0.29
12	4	4	4	0	5.74	6.28	**6.35**	0.28
12	4	4	4	0.75	5.74	6.29	**6.39**	0.29
12	4	16	16	0	5.60	6.54	**6.57**	0.29
12	4	16	16	0.75	5.60	6.63	**6.69**	0.31
14	4	0	0	—	6.97	7.90	**7.97**	0.17
14	4	1	1	0	6.95	7.89	**7.97**	0.17
14	4	1	1	0.75	6.95	7.89	**7.97**	0.18
14	4	4	4	0	6.91	7.89	**7.95**	0.18
14	4	4	4	0.75	6.91	7.87	**7.97**	0.19
14	4	16	16	0	6.78	7.96	**8.00**	0.22
14	4	16	16	0.75	6.78	7.99	**8.08**	0.23

In cases with $\left(\mu_{1},\mu_{2}\right)$ equal to (10,2) or (12,2) and low prior variance, the FS design is best—but it is substantially inferior to the FF and AE designs in other situations. We conclude that the FS design option should only be considered if there is a strong prior belief that the new treatment will offer little or no benefit to subpopulation ${𝒮}_{2}$.

For the cases in Table 4, the AE design has higher expected gain than the FF design (with the exception of a couple of cases where the two designs have almost equal expected gain). However, we have failed to find an example where the AE design is vastly superior to both the FS and FF designs: the example in Table 3 with $\left(\mu_{1},\mu_{2})=(14,2\right)$ and $\sigma_{1}^{2}=\sigma_{2}^{2}=25$ and the examples in Table 4 with $\left(\mu_{1},\mu_{2})=(12,2\right)$ and $\sigma_{1}^{2}=\sigma_{2}^{2}=16$ have the highest difference in expected gains in favor of the AE design. One may also argue from the values of $P({ℛ}_{1})$ and $P({ℛ}_{3})$ in Tables 2 and 3 that the AE design shows greater selectivity and is less likely to conclude the new treatment is beneficial to the full population when the treatment effect in ${𝒮}_{2}$ is small or absent altogether.

### 6.3 Adjusting other design parameters

When planning an enrichment trial it is natural to investigate all design parameters and, where possible, optimise their values. Here we consider the timing of the interim analysis at which the decision to enrich may be taken but we note that a similar approach can be taken in setting other design features. Suppose, with the problem formulation described above, we wish to find the best value of τ when the prior distribution of $\left(\theta_{1},\theta_{2}\right)$ is given by $\mu_{1}=12$, $\mu_{2}=4$, $\sigma_{1}^{2}=\sigma_{2}^{2}=25$ and ρ=0.75. We have applied our methods to find the Bayes optimal design for different values of τ. Here we used weights $w_{1}=\sqrt{\tau }$ and $w_{2}=\sqrt{1-\tau }$ in the combination test to account for the different sample sizes before and after the interim analysis. Table 5 shows properties of designs with values of τ ranging from 0.1 to 0.9. We see that our earlier choice of τ=0.5 yields the highest expected gain of 6.91, but designs with τ between 0.3 and 0.6 are very close to this optimum. As τ increases from 0.1 to 0.7, the probability of enriching the trial increases. This is in keeping with the information in Table 3 that the FF design is superior to the FS design, so a certain amount of data is needed to show that enrichment is the better option in a particular trial. We have seen similar results in other examples where the the FF design is superior to the FS design: AE designs with a range of τ values perform well, as long as τ is high enough to give enough information to make an informed decision about enrichment.

**TABLE 5:** Properties of the optimal adaptive enrichment (AE) design for different timings of the interim analysis τ when θ has the prior distribution given by (13) with $\mu_{1}=12$, $\mu_{2}=4$, $\sigma_{1}^{2}=\sigma_{2}^{2}=25,$ and ρ=0.75. The interim analysis takes place after a fraction τ of the total sample has been observed

τ	$P({ℛ}_{1})$	$P({ℛ}_{3})$	*P*(Enrich)	E{G(θ,X)}
0.1	0.14	0.58	0.13	6.84
0.2	0.17	0.56	0.23	6.87
0.3	0.19	0.55	0.28	6.89
0.4	0.19	0.55	0.31	**6.91**
0.5	0.18	0.55	0.33	**6.91**
0.6	0.17	0.56	0.34	6.89
0.7	0.15	0.57	0.34	6.88
0.8	0.13	0.58	0.32	6.87
0.9	0.11	0.59	0.27	6.85

A somewhat different pattern is seen in scenarios where the FS design gives a high expected gain. Suppose the prior distribution for $\left(\theta_{1},\theta_{2}\right)$ has $\mu_{1}=12$, $\mu_{2}=2$, $\sigma_{1}^{2}=\sigma_{2}^{2}=4$ and ρ=0.75. We saw in Table 4 that the FS design has higher expected gain than both the FF design and the optimal AE design with τ=0.5. Table 6 shows results for optimal AE designs with different values of τ.

**TABLE 6:** Properties of the optimal adaptive enrichment (AE) design for different timings of the interim analysis when θ has the prior distribution given by (13) with $\mu_{1}=12$, $\mu_{2}=2$, $\sigma_{1}^{2}=\sigma_{2}^{2}=4,$ and ρ=0.75. The interim analysis takes place after a fraction τ of the total sample has been observed

τ	$P({ℛ}_{1})$	$P({ℛ}_{3})$	*P*(Enrich)	E{G(θ,X)}
0.1	0.69	0.21	0.72	5.75
0.2	0.60	0.28	0.64	**5.76**
0.3	0.53	0.33	0.59	5.75
0.4	0.48	0.37	0.55	5.72
0.5	0.42	0.41	0.53	5.67
0.6	0.37	0.44	0.49	5.61
0.7	0.32	0.47	0.45	5.55
0.8	0.27	0.51	0.41	5.48
0.9	0.15	0.54	0.36	5.39

Since we have used weights $w_{1}=\sqrt{\tau }$ and $w_{2}=\sqrt{1-\tau }$ in the combination test, as τ decreases toward zero the analysis after enrichment becomes identical to that of the FS design. This explains why the probability of enrichment is high for small values of τ and the expected gain is very close to that of the FS design. In fact, the optimal AE designs with τ=0.1, 0.2 and 0.3 have marginally higher expected gain than the FS design. Thus, an adaptive design with an early interim analysis could be a suitable choice if investigators are reluctant to restrict attention to subpopulation ${𝒮}_{1}$ from the outset.

### 6.4 Effect of the subpopulation size

In all of our examples so far, the subpopulation ${𝒮}_{1}$ has represented half of the total population. The size of the specified subpopulation is a feature of the study and not a parameter that can be controlled. Table 7 shows the effect of the subpopulation size on the relative performance of different designs. In this example, the prior distribution for $\left(\theta_{1},\theta_{2}\right)$ has $\mu_{1}=14$, $\mu_{2}=2$, $\sigma_{1}^{2}=\sigma_{2}^{2}=25$ and ρ=0.75, and we saw in Table 3 that the optimal AE design is the best option when λ=0.5. The results in Table 7 show that the optimal AE design remains superior to both the FF and FS designs across the whole range of λ values from 0.1 to 0.9.

**TABLE 7:** Properties of fixed subpopulation (FS), fixed full population (FF), and optimal adaptive enrichment (AE) designs for different subpopulation sizes when θ has the prior distribution given by (13) with $\mu_{1}=14$, $\mu_{2}=2$, $\sigma_{1}^{2}=\sigma_{2}^{2}=25,$ and ρ=0.75. The subpopulation ${𝒮}_{2}$ represents a fraction λ of the total population

λ	E{G(θ,X)}			*P*(Enrich)
	FS	FF	AE	for AE
0.1	1.35	2.44	**2.61**	0.56
0.2	2.69	3.58	**3.87**	0.53
0.3	4.04	4.85	**5.14**	0.49
0.4	5.38	6.11	**6.36**	0.44
0.5	6.72	7.33	**7.53**	0.44
0.6	8.06	8.53	**8.71**	0.40
0.7	9.42	9.73	**9.87**	0.39
0.8	10.77	10.93	**11.03**	0.38
0.9	12.11	12.13	**12.18**	0.38

For each design, the expected gain for all designs increases with λ as the fraction of the population in which the treatment effect is $\theta_{1}$ becomes larger. The margin of superiority of the AE design over the FF design is largest for λ=0.2 and λ=0.3. The reasons behind this are quite complex. The potential benefits of adaptive enrichment are small when λ is close to zero or 1 and one of the subpopulations forms a large fraction of the total population. Also, the interim estimate of $\theta_{1}$ has a high variance when λ is small and the estimate of $\theta_{2}$ has a high variance when λ is large, reducing the information available when making the interim decision. Nevertheless, it is clear from this example that adaptive enrichment can be of benefit over a wide range of subpopulation sizes.

## 7 DISCUSSION

We have considered adaptive trial designs for testing the efficacy of a new treatment when a prespecified subpopulation is deemed particularly likely to benefit from the new treatment. The methods we have presented facilitate calculation of the Bayes optimal rule for deciding whether to enrich in a design where the familywise type I error rate is controlled by a closed testing procedure and combination test. Since this calculation relies on Monte Carlo simulation to determine the optimum decision at all possible values of $\left({\hat{\theta }}_{1}^{\left(1\right)},{\hat{\theta }}_{2}^{\left(1\right)}\right)$, efficient calculation is crucial. We achieve this by use of an algorithm that makes intensive computations along a one‐dimensional strip of $\left({\hat{\theta }}_{1}^{\left(1\right)},{\hat{\theta }}_{2}^{\left(1\right)}\right)$ values, rather than on a fully two‐dimensional grid. The use of simulation means that this approach is highly flexible and may be applied just as easily with other forms of closed testing procedure or combination test, or with different definitions of the final gain function.

Our study of a wide range of examples supports clear conclusions about the benefits of adaptive enrichment designs. If investigators are willing to use either the FF (Fixed Full population) or FS (Fixed subpopulation) design, the additional benefits of an adaptive enrichment design are at best modest for the gain function we have considered. However, the FS design may not be a realistic option: there could be differing opinions about the likely treatment effect in the subpopulation ${𝒮}_{2}$ or, within the wider development program, there may be good reasons for wanting to learn about the new treatment's efficacy in the full population. Then, if the FS design is not an option, there are plausible prior distributions for θ under which the AE is clearly superior to the FF design.

A positive feature of AE design that is not captured in our gain function is its selectivity. Suppose $\theta_{1}$ is high but $\theta_{2}$ is close to zero. If rejection of *H*_03_: $\theta_{3}\leq 0$ leads to the new treatment being made available to the full patient population, it would be given to patients in ${𝒮}_{2}$ for whom the control treatment is just as good. If $\theta_{2}=0$, the term $\theta_{3}{ℛ}_{3}$ in the gain function (8) is equal to $\lambda \theta_{1}{ℛ}_{3}$ and this neither rewards nor penalizes giving the new treatment to patients in ${𝒮}_{2}$. The results in Tables 2 and 3 show the AE design to have higher values of $P({ℛ}_{1})$ and lower values of $P({ℛ}_{3})$ compared to the FF design, indicating that when $\theta_{2}$ is low the AE design is more likely to find a treatment effect only in ${𝒮}_{1}$.

Our results have illustrated a general weakness of adaptive designs that decisions about adaptation are based on interim data which provide only limited information about the true treatment effects. The results in Table 2 for the FS and FF designs show clear benefits to drawing patients from the most appropriate subgroups when the value of θ is known. However, in the examples of Table 3 and the examples with higher prior variances in Table 4the AE designs must make enrichment decisions under highly variable posterior distributions of θ at the interim analysis. A possible remedy to this problem in making the enrichment decision is to use additional information from other endpoints or biomarkers that can be assumed to respond in the same way as the primary endpoint to the treatments under investigation.

We have presented methods for a study in which there is just one subpopulation of special interest. These methods can be generalized to the design of trials with multiple subpopulations, possibly nested with the treatment effect increasing as the size of the subpopulation decreases. Then, given a multiple testing procedure that controls FWER, a suitably defined gain function and a prior distribution for the vector of treatment effects, our simulation‐based approach may be used to find the optimal enrichment decision at an interim analysis. However, more computation will be needed to find the full optimal design as the dimensionality of the problem increases with the number of subpopulations.

The gain function (8) may be adapted to reflect the process of drug approval. Suppose, for example, *H*_03_: $\theta_{3}\leq 0$ is rejected on the strength of a large positive estimate of $\theta_{1}$ and a much smaller estimate for $\theta_{2}$. While a regulator may not require formal rejection of the null hypothesis *H*_02_: $\theta_{2}\leq 0$ at the 0.025 significance level, some minimum threshold for an estimate ${\hat{\theta }}_{2}$ may be required in order for the treatment to be approved for the full population, and for health care providers to agree to pay for this treatment. Such a requirement can be reflected in the gain function G(θ,X), where the data in *X* includes estimates of $\theta_{1}$ and $\theta_{2}$. Rather than stipulate a particular gain function for all applications, we recommend that investigators determine the appropriate gain function for their specific trial, then our methods can be used to optimize over adaptive enrichment designs and to compare the resulting design with other, nonadaptive options.

## Supporting information

Data S1. Functions

Data S2. Generate results

## Figures and Tables

**FIGURE 3:**
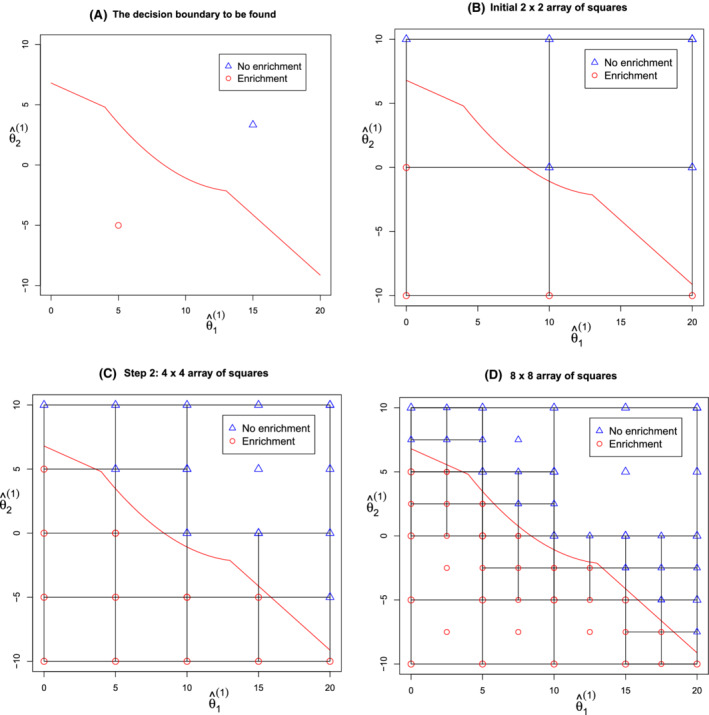
Computation of an optimal decision rule [Color figure can be viewed at wileyonlinelibrary.com]

**FIGURE 4:**
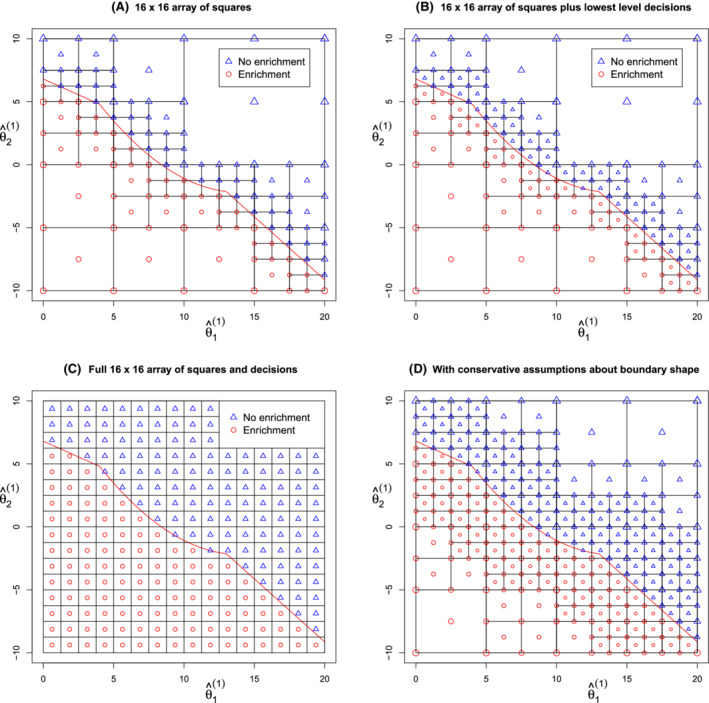
Computation of an optimal decision rule [Color figure can be viewed at wileyonlinelibrary.com]

## Appendix A Strong control of FWER implies a closed testing procedure

### A.1

Suppose a multiple testing procedure 𝒫 with *n* null hypotheses provides strong control of the FWER at level α. We shall show that 𝒫 can be represented as a closed testing procedure 𝒞. Suppose the null hypotheses are stated in terms of a parameter vector θ, then strong control of the FWER implies that

(A1) $$P_{\theta }(\text{Reject at least one true null hypothesis})\leq \alpha \;\text{for all}\;\theta .$$

Suppose the *i*th null hypothesis is *H*_0*i*_: $\theta \in A_{i}$. Denote the observed data by *X* and suppose 𝒫 rejects *H*_0*i*_ if $X\in \xi_{i}$. We shall use the rejection regions $\xi_{i}$ to define a closed testing procedure 𝒞 which gives the same overall decisions as 𝒫.

We first define level α tests of the individual hypotheses *H*_01_, …, *H*_0*n*_. For each *i* ∈ 1, … , *n*, the test of *H*_0*i*_ rejects its null hypothesis if and only if $X\in \xi_{i}$. To see that this gives a level α test of *H*_0*i*_, suppose $\theta \in A_{i}$, then

$$P_{\theta }(\text{Reject}\;H_{0i})=P_{\theta }(X\in \xi_{i})\leq P_{\theta }(\text{Reject at least one true null hypothesis})\leq \alpha ,$$

by applying (A1) with $\theta \in A_{i}$.

Now consider an intersection hypothesis *H*_*I*_ = ∩_*i* ∈ *I*_*H*_0*i*_, where *I* is a subset of {1, … , *n*}. Our level α test of *H*_*I*_, rejects *H*_*I*_ if

$$X\in \cup_{i\in I}\;\xi_{i}.$$

To see this gives a level α test of *H*_*I*_, suppose *H*_*I*_ is true, so $\theta \in \cap_{i\in I}A_{i}$, then

$$P_{\theta }(\text{Reject}\;H_{I})=P_{\theta }(X\in \cup_{i\in I}\;\xi_{i})\leq P_{\theta }(\text{Reject at least one true null hypothesis})\leq \alpha ,$$

by applying (A1) with $\theta \in \cap_{i\in I}A_{i}$.

The closed testing procedure 𝒞 is formed by combining the level α tests of individual and intersection hypotheses in the usual way. Thus, the null hypothesis *H*_0*i*_ is rejected overall if the level α tests reject *H*_0*i*_ and every *H*_*I*_ for which *i* ∈ *I*. It is easy to check that the procedure 𝒞 rejects *H*_0*i*_ overall if and only if $X\in \xi_{i}$, and thus the two procedures 𝒞 and 𝒫 always reject exactly the same set of hypotheses.

Although the above construction is quite simple, we are not aware that this result has been noted previously. An implication in our application is that we lose no generality by restricting attention to methods based on closed testing procedures. Of course, the choice of closed testing procedure remains. In our case, it is natural to base the level α test of *H*_01_ on ${\hat{\theta }}_{1}^{\left(1\right)}$ and ${\hat{\theta }}_{1}^{\left(2\right)}$ and the level α test of *H*_03_ on ${\hat{\theta }}_{3}^{\left(1\right)}$ and ${\hat{\theta }}_{3}^{\left(2\right)}$, so we see it is the method of testing the intersection hypothesis *H*_01_ ∩ *H*_03_ that may merit further investigation.

## Appendix B Derivation of the optimal decision rule

### B.1

We illustrate the details of our computational method in an example where the decision rule being sought is that depicted in Figure 3A. In finding this rule we start by defining a region *A* in which $\left({\hat{\theta }}_{1}^{\left(1\right)},{\hat{\theta }}_{2}^{\left(1\right)}\right)$ will lie with very high probability: in this example we have taken *A* to be the square (0, 20) × (− 10, 10). We subdivide *A* into four smaller squares and find the optimal decision at each of the nine vertices of these squares, giving the results shown in Figure 3B. We proceed on the assumption that if a certain decision is optimal at $\left({\hat{\theta }}_{1}^{\left(1\right)},{\hat{\theta }}_{2}^{\left(1\right)})=(a,b\right)$ and $\left({\hat{\theta }}_{1}^{\left(1\right)},{\hat{\theta }}_{2}^{\left(1\right)})=(a,c\right)$, where *b* < *c*, then the same decision is optimal at all points $\left({\hat{\theta }}_{1}^{\left(1\right)},{\hat{\theta }}_{2}^{\left(1\right)})=(a,d\right)$ with *b* < *d* < *c*; similarly if a decision is optimal at $\left({\hat{\theta }}_{1}^{\left(1\right)},{\hat{\theta }}_{2}^{\left(1\right)})=(a,b\right)$ and $\left({\hat{\theta }}_{1}^{\left(1\right)},{\hat{\theta }}_{2}^{\left(1\right)})=(c,b\right)$, where *a* < *c*, we assume this decision is also optimal at $\left({\hat{\theta }}_{1}^{\left(1\right)},{\hat{\theta }}_{2}^{\left(1\right)})=(d,b\right)$ for all *a* < *d* < *c*. Applying this assumption in our example, we see that it is optimal to enrich for all values $\left({\hat{\theta }}_{1}^{\left(1\right)},{\hat{\theta }}_{2}^{\left(1\right)}\right)$ in the top right‐hand square, so we record this conclusion and make no further calculations for points in this square. The other three squares need further work: we subdivide each of these into four smaller squares and find the optimal decision at each new vertex. The results of these steps are presented in Figure 3C.

We continue the search iteratively, halving the size of the smallest squares at each step. In the next iteration for our example, we note that five of the 12 small squares in Figure 3C have the same optimal decision at all four vertices and we allocate this decision to the whole square. We subdivide the other seven squares and compute optimal decisions at the new vertices. The information after this step is depicted in Figure 3D. Repeating the same steps in the next iteration produces the results shown in Figure 4A.

If our target is to specify optimal decisions on a 16 × 16, this is the final iteration. To complete the process, we find the optimal decision associated with each of the smallest squares: if the optimal decision is the same at all four vertices this decision is assigned to the square; if not, we find the optimal decision at the square's center point and define this to be the decision for the whole square. Figure 4B shows the results of this last step, while Figure 4C presents the same set of conclusions using the full 16 × 16 grid.

Analysing this algorithm in the most demanding case when the decision boundary is at an angle of 45°, we find the optimal decision has to be computed at about 14*n* points in order to determine optimal decisions on an array of *n* × *n* small squares. A key point here is that the amount of computation is of order *n*, even though there are *n*^2^ small squares at the finest level. Since we need to conduct a large number of simulations in finding the optimal decision for each value of $\left({\hat{\theta }}_{1}^{\left(1\right)},{\hat{\theta }}_{2}^{\left(1\right)}\right)$, the computational load can still be high—but it is feasible. In our examples we found optimal decisions on a 2^8^ × 2^8^ or 2^9^ × 2^9^ array, using samples of size 10^5^ or 10^6^ from the posterior distribution of θ in finding the optimal decision at each $x_{1}=({\hat{\theta }}_{1}^{\left(1\right)},{\hat{\theta }}_{2}^{\left(1\right)})$.

In the examples we have studied, it has usually been clear from the results that the optimal decision function has the assumed monotonicity property. However, it is possible for this assumption to fail. In that case, the decision boundary may cross one edge of a square twice, then having the same optimal decision at all four vertices of that square does not necessarily mean this decision is optimal throughout the square. In a more conservative version of our algorithm, which guards against this eventuality, we require the same decision to be optimal at all 16 vertices of a 3 × 3 grid of squares before concluding this decision to be optimal over the whole of the central square. The additional computations needed when this approach is followed in our example are illustrated in Figure 4D. In general, this conservative approach requires approximately twice the total computation time.
